# Spatial electricity market data for the power system of Kazakhstan

**DOI:** 10.1016/j.dib.2019.103781

**Published:** 2019-02-23

**Authors:** Makpal Assembayeva, Jonas Egerer, Roman Mendelevitch, Nurkhat Zhakiyev

**Affiliations:** a Nazarbayev University, National Laboratory Astana, Laboratory Energy, Ecology and Climate, 53 Kabanbay Batyr Ave., 010000 Astana, Kazakhstan; b Technische Universität Berlin, Workgroup for Infrastructure Policy (WIP), Strasse des 17. Juni 135, 10623 Berlin, Germany; c German Institute for Economic Research (DIW Berlin), Dept. Energy, Transport, Environment, Mohrenstr. 58, 10117 Berlin, Germany; d Humboldt Universität zu Berlin, Resource Economics Group, Unter den Linden 6, 10999 Berlin, Germany; e Friedrich-Alexander-Universität Erlangen-Nürnberg, Chair of Economic Theory, Chair of Industrial Organization and Energy Markets, Energie Campus Nürnberg (EnCN), Lange Gasse 20, 90403 Nürnberg, Germany; f Nazarbayev University, National Analytical Center, Center of Macroeconomics and Finance, 53 Kabanbay Batyr Ave., 010000 Astana, Kazakhstan

## Abstract

The data presented in this article are related to the research article “A spatial electricity market model for the power system: The Kazakhstan case study” (M. Assembayeva et al. 2018). This data article presents information on network topology and characteristics, demand variation and distribution, technical and economic parameters for conventional and renewable generation, as well as availability time series, and imports and exports. The dataset is made publically available to allow for more and independent analysis of this emerging energy market.

**Table:** Specifications table

Subject area	*Energy Economics*
More specific subject area	*Technical and Economic Data on the Kazakh Electricity System*
Type of data	*Tables, graphs, data file*
How data was acquired	*An extensive compilation of data from various sources in literature, including translation from Russian, expert knowledge; google maps was used to identify geo-coordinates of transmission nodes and power plants, line length was determined using Qgis*
Data format	*Processed*
Experimental factors	*Network layout was taken from KEGOC topology map but geocoded using google maps*
Experimental features	*Data on power plant location, capacity, efficiency and age structure was retrieved from various public sources and reconfirmed with expert knowledge*
Data source location	*Kazakhstan (N 48.005284 E 66.9045434)*
Data accessibility	*Data is with this article and included in the accompanying excel file*
Related research article	M. Assembayeva, J. Egerer, R. Mendelevitch, and N. Zhakiyev, A spatial electricity market model for the power system: The Kazakhstan case study, Energy. 2018, vol. 149, pp. 762–778 [1]

**Table:** 

**Value of the data** • The data serves as a starting point for a better understanding of the technical and spatial system characteristics and the underlying energy economics of the Kazakh electricity system • The data can be used for models with a techno-economic focus to inform the public and policymakers and foster a transparent discussion on future power system restructuring • The references present the status-quo on publically available data for the electricity system of Kazakhstan and highlight where additional public data could provide additional benefits • The presented data is valuable input not only to other electricity market and investment modelers that do not speak Russian, but it also provides vital data for energy system and electrical engineering models that focus on Kazakhstan

## 1 Data

This article explains the input data (excel file) which was used for a detailed analysis of the electricity system of [1] and to provide additional information on data acquisition and compilation. A combination of quantitative and qualitative approaches was used in the data analysis of network topology and characteristics, demand variation and distribution, technical and economic parameters for conventional and renewable generation, as well as availability time series, and imports and exports.

The data file has five spreadsheets and is structured as follows: The spreadsheet General Information contains definitions of sets with a short description and information on related entries as well as a list of network nodes with additional information. The spreadsheet LINES contains technical parameters of the individual network lines and the network topology based on [2], [3], [4]. The spreadsheet DEMAND contains hourly electricity demand data for a winter and a summer week in Kazakhstan, allocated to the nodes defined on the General Information spreadsheet with data based on [5], [6], [7], [8]; import and export flow are also reported here. The spreadsheet PLANTS lists power plants on the block level and their main technical and economic characteristics based on data from Refs. [9], [10], [11], [12], [13]. The spreadsheet PV_WP provide corresponding regional time series for the availability of wind and PV energy sources based on data from Refs. [14], [15].

## 2 Experimental design, material and methods

### 2.1 Data of Kazakhstan's transmission system

**Fig. 1:**
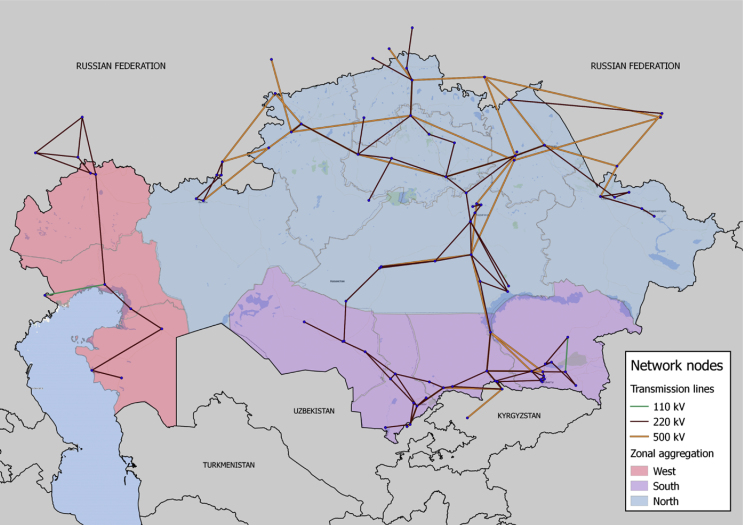
Electricity map of Kazakhstan (2015). Source: Own figure based on [1].

Data on Kazakhstan's transmission system in spreadsheet *LINES* depicts the state of the system in 2015. It includes details on the location of the substations and the topology and technical parameters for the high-voltage transmissions lines. Fig. 1 presents the topology of the grid of Kazakhstan according to the data provided by the transmission system operator KEGOC and verified in individual desk research to identify location and interlinkage in the system.

The country's high voltage transmission system consists of 310 lines (35–1150kV). The dataset provides information on 193 lines, as it only considers the range of 220–1150 kV plus some additional 110 kV lines to specify the representation of flows in the region of Almaty and Atyrau The 1150 kV lines are now operated as 500 kV lines. Each line is identified through a unique identifier referring to its voltage level and its location.

Technical characteristics of the transmission lines included in the dataset such as voltage level, number of circuits, thermal power flow limits, and loss factors have been taken [2], [3], [4]. The line length is calculated based on geo-information on the substations using qgis.

### 2.2 Data on electricity demand

Nodal hourly demand data for one summer and one winter week is reported in the spreadsheet *DEMAND*. The respective values are derived as follows:

1) Two types of electricity demand are considered: residential and industrial
2) Annual Industrial demand for 2015 is known for the 15 biggest companies [7]. Their demand is allocated to the closest substation and assumed to have a flat profile.
3) Residential demand is distributed to 24 residential centers (cities with a population above 70,000) according to population statistics [8].
4) Residential demand is assumed to fill the gap between industrial demand and data on the hourly national demand profile for 2013 [5]. The national demand profile is distributed to the regional level using [5]. The resulting demand levels are given in Table 1.

**Table 1:** Calculated nodal demand for 2013.

Node	Region	Residential demand	Industry demand	Substation	Annual nodal demand [TWh]
N0000	Akmola	Kokshetau		1150 Kokshetauskaya	0.73
N0001	Akmola	Astana	NC KTZH JSC^a^	500 CGPP	5.13
N0007	North Kazakhstan	Petropavlovsk			3.38
N1002	West Kazakhstan	Uralsk		220 ″Uralskaya"	1.66
N1003	Aktobe	Aktobe	AZF TNK Kazchrome JSC (Aktobe)	220 ″Aktyubinskaya"	3.94
N2004	Almaty	Almaty	NC KTZH JSC^a^	220 Robot	0.12
N2008	Almaty	Almaty		220 Taugul	0.62
N2009	Almaty	Almaty		220 Eremensay	1.86
N2011	Almaty	Almaty		220 SS#7	0.41
N2012	Almaty	Almaty			2.89
N2013	Almaty	Almaty			3.09
N2014	Almaty	Taldykorgan			0.79
N3002	East Kazakhstan	Ust-Kamenogorsk	KazZinc LLP, UK TMK JSC (Ust-Kamenogorsk titanium and magnesium plant)	220 #7	8.63
N4000	Mangystau	Zhanaozen			1.62
N4001	Mangystau	Aktau			2.84
N4003	Atyrau		Tengizchevroil LLP	220 Tengiz	2.28
N4004	Atyrau	Atyrau			1.62
N5000	Kostanay	Kostanay	NC KTZH JSC^a^	1151 ″Kostanaiskaya"	2.10
N5001	Kostanay	Rudnyi	Sokolov-Sarybai Mining Production Association (SSGPO) JSC	500 ″Sokol"	3.43
N6000	Pavlodar	Ekibastuze	NC KTZH JSC^a^	1150 ″Ekibastuzskaya"	2.18
N6001	Pavlodar	Pavlodar	Pavlodar Aluminum Plant JSC, Kazakhstan electrolysis plant JSC, Aksu Ferroalloy Plant JSC	220 EEK - Aksu, AZF	15.35
N7004	Karaganda		Arselor Mittal Temirtau JSC	Metallurgicheskaya	3.86
N7005	Karaganda	Karaganda	K. Satpayev channel RGP		5.05
N7006	Karaganda		NC KTZH JSC^a^		0.91
N7008	Karaganda	Balchash	Corporation Kazakhmys LLP Balkhash	Balchashskaya	1.40
N7010	Karaganda	Zhezkazgan	Corporation Kazakhmys LLP Zhezkazgan	Zhezkazgan	2.38
N7012	Karaganda	Temirtau			1.77
N8000	Zhambyl	Taraz	Taraz Metallurgical Plant LLP, Kazphosphate LLP	500 Zhambyl (Taraz)	3.49
N8005	South Kazakhstan	Turkestan		220 Mirgalimsay	0.63
N8008	South Kazakhstan	Shymkent	NC KTZH JSC^a^		3.36
N8010	Kyzylorda	Kyzylorda		KyzylOrda	1.20
N8012	Kyzylorda	Baykonur			0.28
N8014	Zhambyl		NC KTZH JSC^a^	Chu 500	0.17

a NC KTZH JSC - National Company Kazakhstan Temir Zholy JSC – demand is adjusted by regions according to the data of railway electrification.
5) To arrive at final values for 2015, residential demand is scaled up so that total regional demand matches figure reported for 2015 in Ref. [7].

### 2.3 Data on electricity generation units

**Fig. 2:**
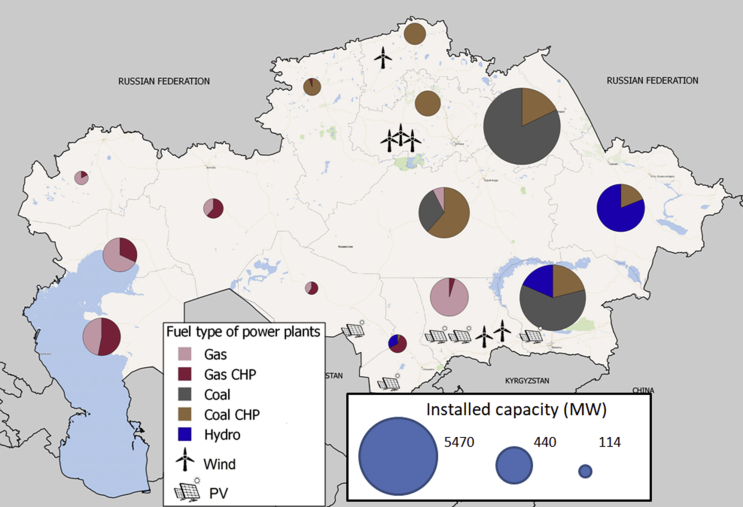
Conventional and renewable power plants capacity in Kazakhstan in 2015.Source: Own figure based on [1].

The spreadsheet PLANTS provides information about generation capacities on the unit level: geographical location, fuel type, combined heat and power (CHP) ability, year of installation, net nameplate capacity and efficiency, as well as marginal cost, seasonal minimum load factors, and availability factors. Data on the individual block level was gathered in an individual desk review building on data from Ref. [13]. It is gathered from various sources, translated and verified with experts on the Kazakh electricity system. Fig. 2 shows the location and size of generation capacity in Kazakhstan by generation technology. It highlights the regional differences and provides an intuition about the distribution of resources and natural potentials (coal transports from North to South by rail). Table 2 provides an overview of the data in an aggregated fashion.

**Table 2:** Aggregated data on conventional power plants.

Fuel	Techno-logy	Purpose	Capacity (MW)	Start year	Average capacity factor (%)^a^	Availability (%)^b^		Average efficiency (%)^c^	Fuel costs (KZT/kW_h_)^d^
						Winter	Summer		
Coal	ST		663–4000	1962–1980	65	0.8–1.0	0.8–1.0	32	0.3–0.5
Coal	ST	CHP	12–1000	1937–2009	57	0.8–1.0	0.5–0.8	42	0.3–2.2
Gas	GT		6–244	1996–2012	58	1.0	0.9–1.0	33	1.8–2.2
Gas	ST		460–1230	1983–2006	28	0.7–1.0	0.5–1.0	34	1.8–2.2
Gas	ST	CHP	4–630	1944–1981	42	0.8–1.0	0.5–0.9	44	1.8–2.2
Hydro			2–702	1928–2013	33	0.3–1.0	0.7–1.0	93	

Source: Own table.

a The capacity factors are calculated based on historical data.

b Availability of the power plant fleet is based on historical data for 2013 and calibrated based on the fuel used (coal, natural gas or oil) and the technology (steam or gas turbine). The main sources were official annual reports of power generation companies.

c Efficiency factors are based on own assumptions based on the age and technology of the unit.

d Fuel prices include fuel and transportation cost and are taken from Refs. [11], [12].

For hydropower, seasonal availability factors are calculated based on data provided by Ref. [14]. Time series data for Wind is based on annual generation output of existing units [16] and on historical data on wind speed and solar radiation. To convert to an hourly and nodal wind time series, wind speeds from meteorological stations are transformed using Weibull probability distribution functions. Similarly, data on solar radiation [15] is transformed into a distribution for the network nodes which is then rescaled to an hourly availability between 0 and 1.
