# Supplementary material for: Spatial electricity market data for the power system of Kazakhstan
Source: Data Brief. 2019 Feb 23;23:103781. doi: 10.1016/j.dib.2019.103781 (PMC6661261; doi:10.1016/j.dib.2019.103781)
Supplement: Multimedia component 1 [file mmc1.docx]

We delare no conflict of interest.
